# Inguinal Hernia Repair in a Centenarian

**DOI:** 10.7759/cureus.90326

**Published:** 2025-08-17

**Authors:** Soniya Pateriya, Claudia Corbea, Joseph Russell, Todd Paul Mangione, Wilmer Mata

**Affiliations:** 1 Surgery, Florida International University Herbert Wertheim College of Medicine, Miami, USA; 2 Biophysics and Physiology, Georgetown University, Washington, DC, USA; 3 Internal Medicine, Icahn School of Medicine at Mount Sinai (South Nassau), Oceanside, USA; 4 Surgery, West Kendall Baptist Hospital, Miami, USA

**Keywords:** centenarian, general surgery, inguinal hernia repair, lower abdominal pain, older-aged patients, postoperative complication, preoperative assessment and risk management, revised cardiac risk index, surgery, surgical complication

## Abstract

Centenarians present unique challenges regarding medical care, especially the decision to proceed with surgical intervention. This case report describes the successful surgical management of a 100-year-old man who presented to the emergency room with severe right lower abdominal pain due to a recurrent right inguinal hernia. Despite a moderately complicated past medical history, the patient underwent a successful open right inguinal hernia repair with mesh without complications. Preoperative risk assessments indicated a low cardiac risk, and comprehensive perioperative care facilitated an uneventful recovery. This case underscores the importance of appropriately assessing and optimizing the care for centenarians to safely undergo intermediate-risk surgical interventions, challenging the notion that extreme age alone is a contraindication for surgery.

## Introduction

Reaching the age of 100 years has historically been a medical marvel. With life expectancy increasing in the United States, the number of centenarians (100 years of age or older) is projected to grow from 108,000 in 2024 to 513,000 in 2054 [1]. The rise of the centenarian population is a positive reflection of the improved medical care and quality of life. Still, centenarians and other elderly patients can require additional medical care owing to their changing physiology [2]. Although some centenarians can stave off chronic disease, more than half of the age group develops chronic disease, cognitive impairment, or disability [3].

As a result, surgeons and physicians alike will continue to see a rise in centenarians for whom they will need to decide the need for surgical interventions. Because they often exhibit a complex interplay of age-related frailty, multiple comorbidities, and heightened risk of emergent medical conditions, centenarians and their medical team exercise caution when deciding to proceed with surgical procedures [4]. For instance, one study indicated that the one-year postoperative survival rate of centenarians undergoing surgical interventions was 69% [5]. However, surgical interventions can improve quality of life and extend life. The same study showed that centenarian patients have successfully undergone and survived low- and intermediate-risk procedures without significant complications.

Herein, we report a 100-year-old man who presented to the emergency room with acute right lower abdominal pain. Despite a medical history of chronic illnesses and prior abdominal surgery, the patient successfully underwent an open right inguinal hernia repair, highlighting the feasibility of medical and surgical management in this age group.

## Case presentation

A 100-year-old man presented to the emergency department with worsening right lower abdominal pain. Prior medical history included right inguinal hernia prior inguinal hernia repair, hypertension, gastric ulcer, hyperlipidemia, chronic obstructive pulmonary disease with emphysema, and benign prostatic hyperplasia. At the time of presentation, our patient had recurring and severe right lower quadrant abdominal pain and nausea. He denies vomiting, chest pain, diarrhea, abdominal distention, fever, chills, numbness, or tingling. On examination, he was noted to have a right inguinal hernia, which was reduced by the emergency physician with improvement of the patient's abdominal pain.

**Figure 1:**
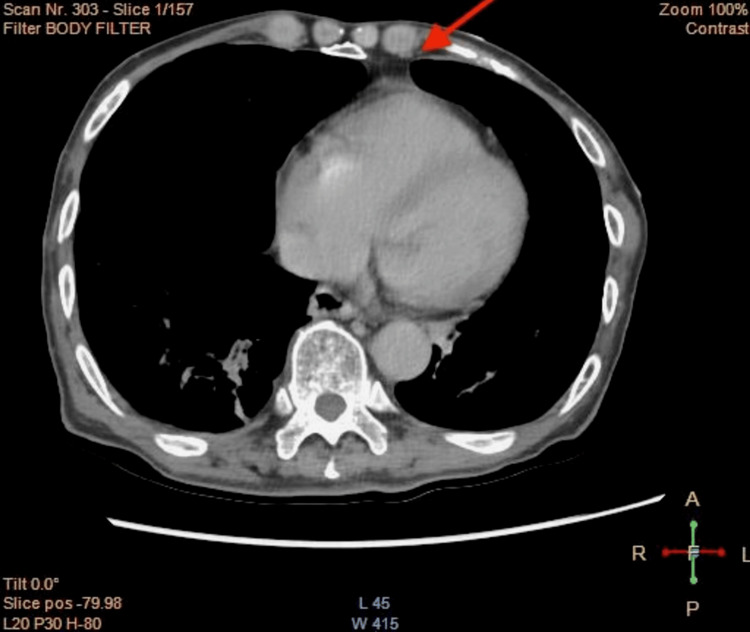
Computed tomography scan with contrast, remarkable for a fluid and fat-containing right inguinal hernia

Upon further questioning, he was able to perform all activities of daily living and lives at home with his family. Prior surgical history was remarkable for right inguinal hernia repair complicated by bowel obstruction, necessitating removal of bowel adhesions in 2016. The patient was hemodynamically stable and afebrile. Laboratory results were notable for chronically low hemoglobin of 12.7 g/dL, normal lactic acid, and negative troponin; comprehensive metabolic panel and urinalysis (UA) were unremarkable. Computed tomography with contrast showed a fluid and fat-containing right inguinal hernia and an atypical bowel pattern without definite obstruction, suggestive of a resolving bowel obstruction (Figure 1).

General surgery was consulted, and hernia repair surgery was recommended, given the patient's intractable abdominal pain and high risk for hernia complications, peritonitis, and death. The patient was assessed to have a Cardiac Risk Index (CRI) of 0 points and a frailty score of 4. He was medically optimized to undergo the intermediate-risk surgery. An open right inguinal hernia repair with mesh was performed without complications. Our patient's postoperative course was unremarkable, and he was successfully discharged.

## Discussion

Centenarians do have a higher risk of surgical complications, especially for intermediate-risk procedures. However, similar case reports and studies show that centenarians can undergo surgical procedures safely (Table 1).

**Table 1:** Comparison of five separate case studies regarding centenarians and surgical interventions

Case series	Number of centenarians	Age (years)	Country	Types of surgical procedures	Mortality data	Complications (postoperative or follow-up)
MacDowall et al. (2021) [4]	25	101 median	Australia	Orthopedic, plastics, maxillofacial, colorectal	0%, in-hospital; 44%, 3-year	Delirium (44%) and constipation (32%)
Cogbill et al. (1992) [5]	25	101.1 mean	United States	Ophthalmologic, pacemaker implantations, hip screw fixations	6%, in-hospital; 31%, 1-year	N/A
Mazzola et al. (2016) [6]	25	101.5 average	Italy	Hip fractures	375-day mean survival postoperatively	Heart failure, stroke, pneumonia, urinary tract infection
López-Torres et al. (2020) [7]	25	101.3 average	Spain	Hip fractures	13.8%, in-hospital; 54.2%, 1-year	Delirium (52%) and urinary retention (27%)
Morice et al. (2017) [8]	25	101.3 average	France	Proximal femur fractures	10.3%, in-hospital; 38.5%, 1-year	Early dislocation (7.7%), surgical site infection (5%), confusion (5%)

In a case report conducted by Giannotti et al., the surgery team was able to perform a lateral decompressive cecostomy procedure on a centenarian patient presenting with ileus secondary to stenotic ascending colon cancer without complications [9]. Badr et al. also reported successfully conducting transcatheter aortic valve implantation in a 103-year-old patient to treat severe symptomatic aortic valve stenosis [10]. Along with our case, there continues to be a growing body of evidence to support low- and intermediate-risk surgeries in centenarians.

Careful preoperative and postoperative assessments are crucial for minimizing risks and improving outcomes. One such preoperative assessment, frailty, is an evidence-based tool that helps predict postoperative mortality, complications, and prolonged length of stay [11]. Surgery and subsequent extended recovery periods can lead to a decline in functional status, making it more difficult for these patients to return home and continue their baseline activities of daily living. Another important preoperative measure is the Cardiac Risk Index, which helps estimate the risk of cardiac complications in patients undergoing non-cardiac surgery [12]. The CRI evaluates six independent predictors of major cardiac complications: high-risk surgery, history of ischemic heart disease, history of heart failure, history of cerebrovascular disease, diabetes requiring insulin therapy, and preoperative serum creatinine > 2 mg/dL. Each criterion met scores one point.

The increased likelihood of post-surgical complications in centenarians causes healthcare providers to thoroughly review the patient's medical history before deeming the patient an appropriate candidate for surgery. In a retrospective study by MacDowall et al., the most common postoperative complications were found to be neurological (i.e., delirium) and gastrointestinal (i.e., constipation). Delirium, in particular, is heavily influenced by factors such as anesthesia, pain medications, and the stress of surgery itself, which can lead to longer hospital stays and increased mortality [13]. Postoperative infections, such as urinary tract and surgical site infections, are also a common area of concern for this population due to their relatively compromised immune system, making them more susceptible [14].

Our centenarian patient presented with abdominal pain secondary to an inguinal hernia. His intractable pain and potential for improved quality of life, coupled with a low CRI, provided a low threshold for surgical intervention. Our patient's tolerance of the intermediate-risk procedure and lack of postoperative complications are similar to previous reports. However, centenarians can present with life-threatening conditions, such as aortic dissection or ischemic bowel, for which surgery may not be an option [4]. Therefore, despite multiple reports of successful cases, physicians and surgeons should continue to use caution and optimize medical care for centenarians when making treatment considerations.

## Conclusions

Centenarians present a complex situation regarding medical care. The decision to proceed with surgical intervention in a centenarian patient can be complicated. Before surgical treatment is pursued, physicians and surgeons should discuss the risks, complications, and outcomes with the patient while also evaluating medical history, laboratory results, imaging, and CRI to optimize medical therapy. Our patient's tolerance of the intermediate-risk procedure and lack of postoperative complications show that centenarians can have remarkable resilience. This case underscores the importance of appropriately assessing and optimizing the care for centenarians to safely undergo intermediate-risk surgical interventions, challenging the notion that older age alone is a contraindication for surgery.
